# The incidence of type 1 diabetes in children under 15 years of age is rising again—a nationwide study

**DOI:** 10.1007/s00431-023-05125-7

**Published:** 2023-08-08

**Authors:** Edna F. Roche, Amanda M. McKenna, Myra O’Regan, Kerry J. Ryder, Helen M. Fitzgerald, Hilary M. C. V. Hoey

**Affiliations:** 1 The Discipline of Paediatrics, School of Medicine, Trinity College Dublin, Dublin, Ireland; 2 The Research and Evidence Office, Health Service Executive, Dublin, Ireland; 3 The Department of Paediatric Growth, Diabetes, and Endocrinology, Children’s Health Ireland (CHI) at, Tallaght University Hospital, Dublin, Ireland

**Keywords:** Type 1 diabetes, Childhood, Incidence rate, Epidemiology

## Abstract

International incidence rates (IRs) and trends of childhood type 1 diabetes (T1D) vary. Recent data from Ireland and other high incidence countries suggested a stabilisation in IRs of T1D in children aged under 15 years. Our primary objective was to report the IR of T1D in children in Ireland from 2019 to 2021 and evaluate if age, sex and season of diagnosis had changed. Incident cases of T1D in those aged under 15 years were identified prospectively by clinicians nationally and reported to the Irish Childhood Diabetes National Register (ICDNR). Following case verification, capture-recapture methodology was applied, and IRs calculated. Numbers of children including age, sex and season of diagnosis per year were evaluated. There were 1027 cases, 542 males (53%). The direct standardised incidence rates (SIRs) increased by 21% overall and were 31.1, 32.2 and 37.6/100,000/year, respectively, with no significant sex difference. The highest IRs were in the 10–14-year category until 2021, then changed to the 5–9-year category (40% of cases). Whilst autumn and winter remain dominant diagnostic seasons, seasonality differed in 2021 with a greater number presenting in spring.

*Conclusion*: The incidence of childhood T1D in Ireland is increasing, observed prior to the COVID-19 pandemic, and shifting to an earlier age at diagnosis for the first time. The pattern of seasonality also appears to have changed. This may reflect an increased severity of diabetes with important implications for healthcare providers.

**Table:** 

**What is Known:** *• Ireland has a very high incidence of T1D in childhood, which had stabilised following a rapid rise, similar to other high incidence countries.* *• The incidence rate is consistently highest in older children (10–14 years).*	
**What is New:** *• Irish IR is no longer stable and has increased again, with the highest incidence occurring in the younger 5-9 age category for the first time.* *• The seasonality of diagnosis has changed during the COVID-19 pandemic years of 2020–2021.*

**What is Known:**

*• Ireland has a very high incidence of T1D in childhood, which had stabilised following a rapid rise, similar to other high incidence countries.*

*• The incidence rate is consistently highest in older children (10–14 years).*

**What is New:**

*• Irish IR is no longer stable and has increased again, with the highest incidence occurring in the younger 5-9 age category for the first time.*

*• The seasonality of diagnosis has changed during the COVID-19 pandemic years of 2020–2021.*

## Introduction

Type 1 diabetes (T1D) is a chronic autoimmune condition which has been shown to be rising globally for many decades, yet the exact reasons for this remain unclear [1, 2]. Reported incidence rates in the literature between regions and countries can be observed in varied states: falling, plateauing or rising [1, 3–11].

During recent decades, Finland has consistently reported the highest incidence rate (IR) of T1D in children, peaking at 64.9/100,000/year in 2006 [12, 13]. Finland’s children experienced a decrease in IRs (overall IR reducing to 52.2/100,000/year in 2015–2018) after appearing to level off during 2003–2018 [1].

A similar trend has been noted in Ireland as in other high incidence countries. Ireland experienced a sharp rise in incidence between 1997 and 2008, followed by an apparent stabilisation of rates [3, 4, 14]. The Western Australian Children’s Diabetes Database noted no significant change in their temporal trends (2003–2016) following a peak in 2003 [3]. A Welsh study on changing incidence looking at data from 1990 to 2019 found that their incidence rates peaked around 2010 at 31.3/100,000/year and then declined [5]. They concluded that the risk of developing T1D under 15 years was not increasing. The country reported to have the second highest IR worldwide is Sweden [13]. Swedish rates were reportedly plateauing in 2011 [6]; however, in a later report, rates were noted to be rising and not stabilising [7].

Most centres reporting persisting increases in their IR of T1D were those with either previously low incidence rates or those recently experiencing economic changes [8–10]. Hong Kong, as a low incidence country, reported increasing rates in children up to 18 years (4.3/100,000/year) detailing data from 2008 to 2017, almost double that of the previous decade although the rate of rise slowed towards the end of the study [11]. However, other centres, such as the Calabria region of Italy, maintained and increased their very high rates, with Calabria showing a marked increase in the last 2 years of their 3-year study (2019–2021) [9]. The German (DPV) registry which collects data on children up to 18 years of age found their IRs increased from 2011 to 2019 and this trend continued into 2020 as predicted [15, 16].

Fluctuation in the incidence of T1D within populations has long been recognised with some centres showing a periodicity in incidence [17]. Monitoring changes in the incidence of T1D internationally is important as it may help shed light on the elusive mechanism(s) triggering this poorly understood disease. The apparent stabilisation (or decline) of incidence rates in a number of high incidence countries, including Ireland, raised the possibility that a saturation point of diabetes had been reached in these populations [1, 3, 5, 14]. However, as Sweden with the second highest incidence of T1D in the world has demonstrated rising incidence rates following a previous plateau, it is important to examine recent data relating to the current status of T1D incidence, particularly in high incidence countries to ascertain if the incidence has remained stable, is falling or is increasing again.

A wide range of environmental triggers, including viruses, obesity and lack of vitamin D, have been postulated to account for these global differences and fluctuating rates [1, 18, 19]. A recent study from the University of Dublin, Trinity College, highlighted that up to one quarter of children living in Dublin (Ireland’s capital city) have low vitamin D levels [20]. Low levels of vitamin D have long been postulated to be associated with T1D progression/development and particularly the north–south gradient in T1D incidence. However, the vitamin D data are somewhat conflicting [21, 22].

A related factor is seasonal variation at diagnosis which is likely influenced by temperature and viruses. Harvey et al. in their 2021 study in Wales (UK) looked at the effect of seasonality and found a greater effect of seasonality at diagnosis with higher rates of diabetes in winter [5]. They noted that their meteorological data showed increasing hours of sunlight since 1980 and postulated that this was associated with stabilisation in their IR due to the effect of sunlight on vitamin D levels [5]. A Swedish study found a stronger association with low mean temperature than hours of sunshine with the development of T1D in children [23].

The primary aim of this study is to determine the incidence rate of T1D in children in Ireland aged under 15 years in the period 2019 to 2021 and compare it with the robust annual incidence data of the Irish Childhood Diabetes National Register (ICDNR) established in 2008, in order to confirm if the previously noted stabilisation of IR over recent years has persisted, reflecting a true stabilisation or whether the incidence is rising again in this high incidence country. Age, sex and season of diagnosis were also evaluated to ascertain if they had changed over the period.

## Materials and methods

New or incident cases of T1D for the period 2019 to 2021 were reported using the established methodology of the Irish Childhood Diabetes National Register (ICDNR), which continues to work closely with all participating centres nationally responsible for the diagnosis and treatment of children with T1D in Ireland. Details regarding the ICDNR have been published previously and are unchanged, apart from the merger of one of the smaller centres with a larger centre in 2019 [14, 24]. Briefly, this involves detailed prospective reporting of incident cases of T1D from all 19 paediatric centres caring for children nationally, case verification and the application of capture-recapture methodology (with the support of the Primary Care Eligibility and Reimbursement Service (PCERS)) [25]. The case definition employed is similar to that of the WHO Diabetes Mondial study and Eurodiab Tiger study group [26, 27]. Inclusion criteria, as in previous years, were physician diagnosed new onset T1D in a child aged under 15 years occurring in the period from January 1st to December 31st and resident in Ireland at the time of diagnosis. The date of diagnosis was taken as the time of first insulin injection. Cases were excluded if children were aged over 15 years or not resident in Ireland at diagnosis, if diabetes was diagnosed outside the study period or if diabetes was not T1D, such as monogenic or type 2 diabetes. Written informed consent was obtained from parent(s) of each child participant. Ethical approval for this study was granted by the SJH/AMNCH Joint Research Ethics Committee in accordance with the Declaration of Helsinki.

The age categories chosen for analysis are 0–4.99 years (under 5), 5–9.99 (5–10 years) and 10–14.99 years (under 15 years). Age and sex category specific incidence rates were calculated using population data from the Central Statistics Office (CSO) [28]. Rates are reported per 100,000 of population for children under 15-year-old (in 5 year groupings) per year. Standardised incidence rates (SIRs) for age and sex were calculated to permit comparison between populations internationally and over time. The direct method of standardisation was employed using a common standard population comprising equal numbers in the six age and sex categories [29]. The results are reported for males, females and all children combined.

Seasons were defined using the meteorological classification of spring (March, April and May); summer (June, July and August); autumn (September, October and November) and winter (December, January and February). This is in line with Europe and the World Meteorological Organisation. Of note the Celtic Irish cultural classification is slightly different wherein each season begins a month before the corresponding meteorological season, i.e. winter commences in November and spring in February [14, 30]. Seasonality data were calculated using the number and percentage of cases diagnosed per season for each year.

Data were analysed using IBM Statistical Package for Social Sciences (SPSS) version 27.0 [31]. Exact confidence intervals for category specific and standardised rates were obtained using the freely available programming language R version 4.2.2 [32]. Chi-square tests and *F*-tests were used where appropriate to compare groups. A Poisson regression was run on the category specific rates as the dependent variable with the log (population) included as an offset to examine the relationship between age category, year and gender. Each variable was treated as a categorical variable.

## Results

In the 3-year period, 2019 to 2021, a total of 1027 children (542 male) aged under 15 years were notified and verified as incident cases of T1D to the ICDNR for calculation of incidence rates. An additional case was identified but subsequently reclassified as having monogenic diabetes and excluded. All paediatric centres nationally notified and offered registration to their eligible children. A number of children, 28 (2.7%), did not complete full registration for a variety of reasons, either they declined or failed to complete the full registration process post-discharge from hospital admission despite multiple reminders. However, anonymous baseline data for gender age and date of diagnosis was provided for all notified cases to enable calculation of IRs and seasonality.

Annual ascertainment rates for the ICDNR were at least 93% in 2019; this is an underestimate, as secondary ascertainment could not be undertaken on 6% of cases where only anonymous baseline data were provided. Thus, the ascertainment rate for 2019 is likely at least 95% and could be as high as 99%. Ascertainment for 2020 and 2021 are incomplete but also will likely be in excess of 93%.

### Age at diagnosis

The mean age of diagnosis fell during the period 2019 to 2021 from 9.4 (3.6) to 9.2 (3.5) and 8.7 (3.6) years, respectively. A one-way analysis of variance showed that there was a significant difference between the average age of diagnosis in the 3 years (*F* = 3.58, df = 2.1024, *p* = 0.028). The Tukey HSD test showed a significant difference between the average age of diagnosis in 2019 and average age of diagnosis in 2021 with the 95% the confidence interval showing the difference ranging from 0.07 to 1.35 years.

### Age and sex categories at diagnosis

In comparison with other years, there was not a statistical difference in the age category at diagnosis between the 3 years (χ^2^ = 8.82, df = 4, *p* = 0.066). The percentage of children diagnosed in the 0–4 and 5–9 age groups increased from 15 to 19% and 34 to 40%, respectively, between 2019 and 2021, whilst the percentage in the 10–14 age category fell from 51 to 40% in the period.

We found no evidence for a difference in the proportions of males and females over the 3 years (χ^2^ = 0.07, df = 2, *p* = 0.97). Overall, there were slightly more males than females (53% to 47%) and the confidence interval for the male to female ratio was 0.98 to 1.25, indicating that there was a not significant difference from a male to female ratio of 1.

### Seasonality at diagnosis

Table 1 presents the season of diagnosis by year. A χ^2^ test showed that there is a significant difference in the seasonal pattern over the years (χ^2^ = 27.44, df = 6, *p* < 0.001). The majority of cases were first diagnosed in autumn and winter seasons in 2019 (58%) and 2020 (60%). This changed in 2021 with less cases diagnosed in autumn and winter (48%), particularly a reduction in cases diagnosed in winter and an increase in children diagnosed in spring (30%).

**Table 1:** Season of diagnosis of type 1 diabetes by year of diagnosis

**Season**	**Year of diagnosis**			
	2019	2020	2021	Overall 2019–2021
	%	%	%	%
Spring	23.6	17.3	30.0	24.0
Summer	18.2	21.9	21.8	20.7
Autumn	25.2	29.2	28.2	27.6
Winter	33.0	31.6	20.0	27.8
Cases (*n*)	318	329	380	1027

χ^2^ = 27.44, df = 6, *p* < 0.001

### Standardised incidence rates

**Fig. 1:**
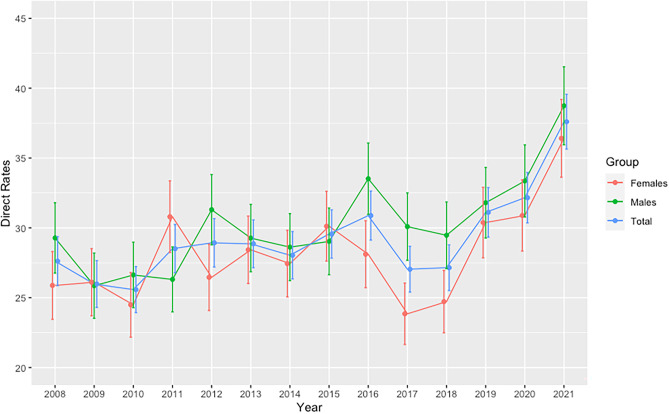
Direct standardised incidence rates of type 1 diabetes 2008 to 2021

Standardised incidence rates were calculated, which allow comparison between populations and over time. Figure 1 shows the trend of the direct standardised incidence rates over the time period 2011 to 2021, overall and also for males and females. The direct standardised rates in the period 2019–2021 were 31.1, 32.2 and 37.6 per 100,000 per year, respectively. The direct standardised rate during this period increased by 21%, slightly more in males (22%) than females (20%). The average percentage increase was 10% per annum overall, 10% in males and 9.5% in females, respectively.

### Crude and category specific incidence rates

The overall crude and category specific incidence rates of T1D by age and sex, which denote the actual experience of the population, are shown in Table 2.

**Table 2:** Overall crude and age and sex category specific incidence rates of type 1 diabetes per 100,000 per year

**Sex and age category**	**Year**	**2019**	**2020**	**2021**
Male 0–4 years	Incidence rate (95% CI)	18.6 (12.6, 26.6)	16.4 (10.7, 24.1)	22 (15.2, 30.8)
	Cases (*n*)	30	26	34
Male 5–9 years	Incidence rate (95% CI)	29.4 (22.0, 38.5)	33.5 (25.5, 43.2)	47.2 (37.5, 58.7)
	Cases (*n*)	53	59	81
Males 10–14 years	Incidence rate (95% CI)	47.4 (37.7, 58.7)	50.2 (40.3, 61.7)	47.0 (37.6, 58.0)
	Cases (*n*)	83	90	86
Female 0–4 years	Incidence rate (95% CI)	11.7 (6.9, 18.5)	16.5 (10.7, 24.4)	26.3 (18.7, 36.0)
	Cases (*n*)	18	25	39
Female 5–9 years	Incidence rate (95% CI)	32.5 (24.6, 42.3)	34.0 (25.7, 44.0)	44.1 (34.5, 55.5)
	Cases (*n*)	56	57	72
Female 10–14 years	Incidence rate (95% CI)	46.9 (37.1, 58.6)	42.2 (33.0, 53.1)	38.9 (30.2, 49.3)
	Cases (*n*)	78	72	68
Male and female 0–4 years	Incidence rate (95% CI)	15.2 (11.2, 20.2)	16.5 (12.3, 21.7)	24.1 (18.9, 30.3)
	Cases (*n*)	48	51	73
Male and female 5–9 years	Incidence rate (95% CI)	30.9 (25.4, 37.3)	33.7 (27.9, 40.4)	45.7 (38.7, 53.5)
	Cases (*n*)	109	116	153
Male and female 10–14 years	Incidence rate (95% CI)	47.2 (40.2, 55.0)	46.3 (39.4, 54.0)	43.0 (36.5, 50.4)
	Cases (*n*)	161	162	154
All males under 15 years	Incidence rate (95% CI)	32.1 (27.4, 37.4)	34.1 (29.2, 39.5)	39.5 (34.2, 45.3)
	Cases (*n*)	166	175	201
All females under 15 years	Incidence rate (95% CI)	30.9 (26.2, 36.2)	31.4 (26.7, 36.8)	36.9 (31.7, 42.7)
	Cases (*n*)	152	154	179
All children under 15 years	Incidence rate (95% CI)	31.5 (28.1, 35.2)	32.8 (29.3, 36.5)	38.2 (34.4, 42.2)
	Cases (*n*)	318	329	380

**Fig. 2:**
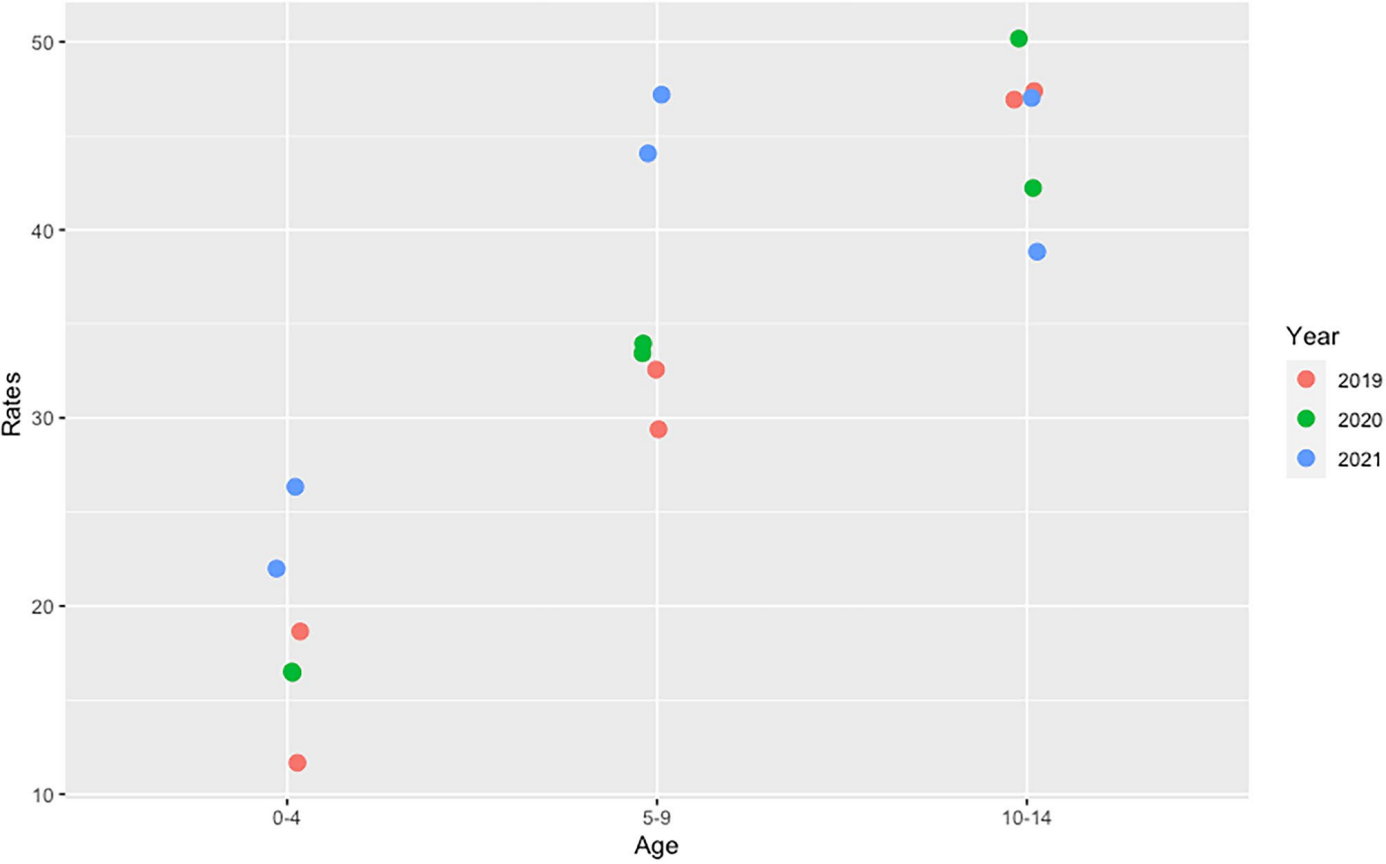
Incidence rates by age (in years) and sex in the period 2019 to 2021 (the two points with the same colour correspond to the males and females)

A Poisson regression was run on the category specific rates as the dependent variable with the log (population) included as an offset for the 3 years (2019–2021). These results should be interpreted with care due to the small number of cases. Three independent variables were included age category, year and sex. Each variable was treated as a categorical variable, i.e. coded using dummy variables. There was no significant sex, sex*age and sex*year effect. It was decided to drop sex from the model. However, there was a significant age*year effect (χ^2^ = 12.6, df = 4, *p* = 0.013) which can be seen clearly from Fig. 2.

## Discussion

This study demonstrates a significantly increased incidence of T1D during the years 2019–2021. The previously observed stabilisation and subsequent minor reduction in the incidence of T1D in the Irish childhood population, suggesting a saturation in childhood diagnoses, has not been maintained [14, 24]. In the period (2019–2021), the direct standardised rate increased by 21% with an average percentage increase of 10%. The trend in the direct standardised incidence rate in the Irish population for the last 14 years (2008–2021) shows a significant increase in the incidence rate (Fig. 1). Of note this increase in the Irish population occurred in 2019 and preceded the COVID-19 pandemic. A similar pattern of apparent stabilisation or fall in incidence rates followed by a subsequent rise in incidence has also been noted by some other high incidence countries, particularly Sweden and more recently reported for Finland [6, 7, 12, 33]. These findings suggest that the environmental pressure that promotes the development of T1D has been maintained, if not increased. However, the variability in T1D incidence continues and not every country or region shows the same pattern. Australia, another high incidence country, has not shown an overall increasing trend thus far [3, 34].

In our population, the increase in incidence has been found in both males and females, although slightly more marked in males (22%) than females (20%). This is unusual for autoimmune conditions which tend to be more prevalent in females. A number of high incidence countries, including Ireland, have long reported a slight excess of males in those diagnosed with T1D in childhood, although not reaching statistical significance [14].

The mean age of diagnosis fell over the 3 years. Since the establishment of the ICDNR and systematic monitoring of incidence rates of T1D in the Irish population, the highest incidence rates have consistently been in the 10–14-year age category. However, in 2021 for the first time this has changed, and the incidence rate has been highest in the 5–9-year age category. Others noting peaks in the 5–9-year age category were Finland, during 2006–2011, Algeria during 2015–2018 and the Calabrian region of Italy during 2019–2021 [9, 10, 12]. This raises a suggestion of a shift to an earlier age at diagnosis in Ireland, which has also been reported in other high incidence countries. Further monitoring is required to see if this effect will be maintained. An earlier age at presentation may also reflect increased environmental pressure and a more aggressive type of diabetes, applying the endotype concept with implications for future therapies, glycaemic control and potentially diabetes-related complications [35].

There have been interesting environmental changes from 2020 onwards with the COVID-19 pandemic that could possibly have an influence on the change in age at diagnosis, for example, in Ireland, children 12 years and older wore face coverings at school from September 2020 whilst younger children did not do so until the end of 2021. This potentially protected older children from viral infections precipitating decompensation of the struggling pancreas with progression to clinical symptoms and presentation whilst leaving younger children more exposed.

### Environmental factors and seasonality

Whilst autumn and winter remain the dominant seasons of diagnosis in children in 2019 and 2020, the seasonality appears to be changing with a greater number presenting in spring in 2021. As previously reported [24], diagnosis in the months of autumn/winter is common in northern hemisphere countries; in Ireland most children are diagnosed in the colder seasons with a winter peak [14, 24].

During 2020–2021, the expected seasonal diagnostic pattern was not observed. Overall findings did not differ from previous register studies in that the autumn–winter numbers remained higher than the spring–summer months with a cumulative figure of 55% diagnosed in autumn and winter. Lower numbers of children were diagnosed during restrictions on society due to the COVID-19 pandemic, most notable, during the first lockdown of spring 2020 and in the winter-spring period of 2020–2021.

The differences in seasonal pattern were likely influenced by societal restrictions and school closures imposed during the COVID-19 pandemic. For example, during school closures the numbers of children diagnosed appeared lower and on relaxation of restrictions the number of cases increased [36]. Schools were closed from March to September 2020 and again from January 2021 to April 2021 [36]. The number of cases diagnosed was reduced in spring 2020, during school closures, they increased slightly over summer 2020 whilst schools remained closed, but at this time there was an increase in societal mixing. Schools re-opened in September 2020 and this coincided with an increase in cases diagnosed in autumn and winter 2020/spring 2021. The number of cases diagnosed in January 2021 was less than usual for the time of year following the “lockdown” post-Christmas 2020. Schools fully re-opened by April 2021 with full relaxation of societal restrictions, and this was reflected in an increased number of cases in spring 2021 [37].

In the period 2019 to 2021, two of these years under study were dominated by the COVID-19 pandemic and whilst cumulative seasonality at diagnosis remained higher (as is usual) in the autumn and winter months, the seasonality was less pronounced. Further monitoring is required to determine if the changes in seasonality persist.

As all children with T1D are admitted to hospital at diagnosis in Ireland, challenges with access to primary care during the COVID-19 pandemic, whilst potentially delaying diagnosis, would not be expected to influence the total number diagnosed with T1D overall. The majority of children with T1D are brought directly to paediatric hospital services by their families.

That the overall rates of T1D are again rising suggests continuing pressure from the yet unidentified environmental factors promoting T1D [2, 18]. Given Ireland’s geographical position, at 53° north of the equator, vitamin D insufficiency is well recognised in our population particularly amongst adolescents and whilst present is unlikely to have worsened markedly in this period. Indeed, in this period routine vitamin D supplementation and rotavirus vaccination have been introduced in infancy, which are postulated to be protective, although studies to date show mixed results [2]. As in other jurisdictions, child overweight and obesity is a major problem in the Irish population potentially increasing pressure on pancreatic insulin production. The rate of child overweight and obesity in Ireland has been stable at approximately 20% from 2015 to 2019 [38]. However, recent unpublished government reports suggest an increase in overweight and obesity of 5% due to the COVID-19 pandemic.

It is hard to interpret the role of infectious agents in this period due to the dramatic societal changes associated with the COVID-19 pandemic for two of these 3 years, with restriction of activities, isolation and “lockdowns” potentially restricting infectious exposures but also the SARS-CoV-2 infection itself which increasingly is thought to be an accelerator of diabetes rather than a precipitant. There may be a role for the “hygiene hypothesis” in this period with reduced infectious exposure promoting the development of T1D although this process would take some years to increase the rates of T1D [39].

The need for improving awareness of childhood diabetes is apparent in this country. As outlined by Ludvigsson [7], increasing rates do not always translate to an increased awareness of the condition. Lack of awareness would potentially lead to a delay in diagnosis and increased severity of presentation but would not influence the overall rate of diabetes. Whilst current studies do not support a direct effect of the SARS-CoV-2 virus infection on the increase in the development of T1D [15, 33], there does appear to be an increase in severity of clinical presentation of T1D due to the effects on the pandemic on healthcare delivery.

Further research is needed relating to the demography and influence of the recent significant increase in immigration in western countries, many of whom have come from countries where there is paucity of data relating to T1D in children and the incidence unknown [40, 41].

Increasing rates of T1D places increasing demands on health services to provide appropriate clinical services to care for children and adolescents with T1D. Where children are diagnosed at an earlier age this further increases their healthcare needs not only due to the challenges of managing T1D in younger children due to their vulnerability to hypoglycaemia and dependency in care but also due to a longer duration of diabetes and potentially greater adverse complications.

A major strength of this study lies in its national coverage, high participation rate and robust methodology unchanged since the development of the ICDNR in 2008. The study is limited by relatively small numbers.

It remains extremely important to continue to monitor trends and variations in the epidemiology of childhood T1D. Health services are under ever increasing strain following the COVID-19 pandemic and in 2022 with recent population growth particularly in the childhood population. In the coming years, important restructuring and reform of the Irish Health services are planned [42]. National registers such as the ICDNR can assist by provision of important robust data which can be used to inform planning services and audit clinical care. The ICDNR can also help shed further light on aetiology through its contribution to international diabetes epidemiology.

In summary, our results show that the incidence of T1D in the Irish childhood population is no longer stable and is rising again with a shift to an earlier age at diagnosis and a suggestion of changing seasonality. This may reflect a more aggressive disease process and has important implications for clinicians and health care providers.

## Data Availability

Not applicable.

## Abbreviations

IR: Incidence rate
ICDNR: Irish Childhood Diabetes National Register
NCHF: National Children’s Hospital Foundation
PCERS: Primary Care Eligibility and Reimbursement Services
SIRs: Standardised incidence rates
T1D: Type 1 diabetes
